# Baseline Lenacapavir Resistance and Capsid Variability in Bulgaria: A Next-Generation Sequencing Study of Newly Diagnosed HIV-1 Infections

**DOI:** 10.3390/pathogens15101011

**Published:** 2026-09-24

**Authors:** Ivailo Alexiev, Aleksandra Partsuneva, Reneta Dimitrova, Asya Kostadinova, Anna Gancheva, Lyubomira Grigorova, Kristina Stoyanova, Maria Nikolova, Radoslava Emilova, Nina Yancheva, Dimitar Strashimirov, Ivan Baltadzhiev, Tsetsa Doychinova, Pavlina Parusheva, Ivelina Todorova

**Affiliations:** 1 National Reference Confirmatory Laboratory of HIV, National Center of Infectious and Parasitic Diseases (NCIPD), 1504 Sofia, Bulgaria; alexandra.partsuneva@gmail.com (A.P.); naydenova.reneta@gmail.com (R.D.);; 2 National Reference Laboratory of Immunology, NCIPD, 1504 Sofia, Bulgariaremilova@ncipd.org (R.E.); 3 University Multi-Profile Hospital for Active Treatment of Infectious and Parasitic Diseases, 1606 Sofia, Bulgaria; 4 Department of Infectious Diseases, Parasitology and Tropical Medicine, MU-Plovdiv, 4002 Plovdiv, Bulgaria; 5 Clinic of Infectious Diseases, University Hospital “Prof. Dr. G. Stranski”, 5800 Pleven, Bulgaria; 6 Clinic of Infectious Diseases, University Hospital “Prof. Dr. St. Kirkovich”, 6003 Stara Zagora, Bulgaria; 7 University Hospital “St. Marina”, 9010 Varna, Bulgaria

**Keywords:** HIV-1, gag, capsid, lenacapavir, capsid inhibitor, drug resistance, next-generation sequencing, molecular epidemiology, phylogenetic analysis, Bulgaria

## Abstract

Lenacapavir, the first approved HIV-1 capsid inhibitor, is now being introduced for both treatment and pre-exposure prophylaxis, yet the capsid-coding region lies outside the pol fragment examined in routine genotypic resistance testing, and baseline data on capsid variability remain scarce, particularly in epidemics dominated by non-B lineages. We applied next-generation sequencing of the HIV-1 gag gene to 103 lenacapavir-naive individuals newly diagnosed in Bulgaria between 2023 and 2025. Nine lineages were identified. Subtype B accounted for 46.6% of the sequences, whereas non-B lineages together predominated (53.4%), led by sub-subtype F1 (13.6%) and CRF01_AE (8.7%); a further 10.7% remained unassigned and grouped predominantly with BF1 recombinant references. The distribution of these lineages differed significantly according to the probable route of transmission (*p* = 0.007). No major lenacapavir resistance-associated mutation was detected (0 of 103; 95% CI 0.0–3.6). Substitutions at position 107 were the only changes observed at a resistance-associated position: T107S in two individuals and a T107T/S mixture in one (2.9% combined). Notably, the two T107S sequences formed a CRF01_AE pair supported by a bootstrap value of 100, indicating that this accessory polymorphism can persist in closely related viruses. All viruses were genotypically predicted to remain susceptible to lenacapavir. These findings establish a molecular baseline for capsid surveillance in Bulgaria, a South-Eastern European country with a genetically diverse HIV-1 epidemic.

## 1. Introduction

Human immunodeficiency virus type 1 (HIV-1) is characterized by an exceptional degree of genetic variability, driven by the error-prone reverse transcriptase, a high replication rate and frequent recombination between co-circulating strains. Group M, which is responsible for the global pandemic, is currently divided into nine subtypes and a growing number of sub-subtypes, together with more than 160 circulating recombinant forms (CRFs) and countless unique recombinant forms (URFs) [1,2]. The distribution of these lineages is markedly heterogeneous between geographical regions and key populations, and recombinants now account for a substantial and increasing share of infections worldwide [3]. This diversity is not merely of taxonomic interest: it may influence the performance of diagnostic and viral load assays, the genetic barrier to resistance and the pathways along which resistance emerges, and it remains a central obstacle to vaccine development [1,2].

The Balkan region, and Bulgaria in particular, has repeatedly been described as an area of unusually high HIV-1 genetic diversity, sustained by continuous viral inflow from both Western and Eastern Europe [4,5]. Molecular studies of the Bulgarian epidemic have documented the co-circulation of subtype B, sub-subtype F1, CRF01_AE, CRF02_AG and several sub-subtypes of subtype A, together with a range of other lineages and recombinants [5,6,7]. These lineages are unevenly distributed across transmission groups: CRF01_AE has been associated with a large outbreak among people who inject drugs (PWID), whereas subtype B has predominated among men who have sex with men (MSM) [8,9]. The neighbouring countries display comparable diversity with distinct national profiles: subtype B predominates in Croatia and Slovenia, although against very different levels of transmitted drug resistance [10,11]; Romania has remained a reservoir of sub-subtype F1 since the paediatric nosocomial epidemic of the late 1980s [12,13]; and the Greek epidemic, historically concentrated among MSM, was reshaped by a large outbreak among PWID in Athens between 2011 and 2013 [14,15]. Transmitted drug resistance has remained comparatively low against this diverse background, and it occurs in clusters spanning different transmission groups and lineages [6,16]. Virtually all of this evidence, however, derives from the pol region, which is the target of routine genotypic resistance testing, and other parts of the genome, including gag, have been studied far less systematically.

The gag gene encodes the Gag polyprotein precursor, which is cleaved by the viral protease during maturation into the matrix (p17), capsid (p24), nucleocapsid (p7) and p6 proteins together with two spacer peptides [17]. The capsid protein is of particular interest. In the mature virion it assembles into the conical core that encloses the viral genome, and it participates in essentially every stage of the replication cycle, including protection of the reverse transcription complex from innate sensors and restriction factors, trafficking to the nucleus, nuclear import of the pre-integration complex, uncoating, integration site selection and, in the late phase, particle assembly and maturation [18,19]. This multifunctional role imposes strong structural constraints on capsid variability, and it also makes the capsid an attractive antiviral target.

Lenacapavir (LEN) is the first approved inhibitor of the HIV-1 capsid. It binds to a hydrophobic pocket at the interface between adjacent capsid monomers and interferes with several stages of the replication cycle, displaying picomolar antiviral potency and no cross-resistance to the existing antiretroviral classes [20,21]. In the CAPELLA trial, lenacapavir added to an optimized background regimen produced durable virological suppression in heavy treatment-experienced individuals with multidrug-resistant HIV-1 [22]. More recently, twice-yearly subcutaneous lenacapavir demonstrated near-complete protection against HIV acquisition in the PURPOSE 1 and PURPOSE 2 trials [23,24], and in 2025 it was approved for pre-exposure prophylaxis by the United States Food and Drug Administration and by the European Medicines Agency and recommended by the World Health Organization as an additional prophylactic option [25]. Lenacapavir is therefore likely to be introduced into European treatment and prevention programs, including in Bulgaria, in the near future.

Resistance to lenacapavir is conferred by a small number of substitutions in the capsid coding region, principally L56I, M66I, Q67H, K70N, N74D, A105E and T107N, which have been selected in vitro and observed in participants experiencing virological failure [20,26]. Several of these substitutions carry a substantial fitness cost, which may limit their persistence and onward transmission. Because the capsid region lies outside the pol fragment amplified in routine genotypic resistance testing, baseline information on capsid variability in untreated populations remains scarce [27]. The surveys published so far have consistently reported a very low prevalence of major lenacapavir resistance-associated mutations among capsid-inhibitor-naive individuals, with polymorphic changes at position 107 being the most frequently observed [27,28,29,30]. Data from settings with a high prevalence of non-B lineages and recombinant forms, however, are still limited, and it remains uncertain to what extent natural capsid variability differs across genetically diverse epidemics.

Next-generation sequencing (NGS) now permits the routine generation of near-full-length or region-specific HIV-1 sequences at high coverage, making it feasible to extend molecular surveillance beyond pol and to characterize the capsid before a capsid inhibitor is introduced into clinical practice. Against this background, the present study applied NGS of the HIV-1 gag gene to samples from individuals newly diagnosed with HIV-1 infection in Bulgaria between 2023 and 2025. The aims were, first, to characterize the genetic diversity and phylogenetic structure of the circulating gag lineages and their distribution across demographic and transmission groups, and second, to determine the prevalence of naturally occurring capsid substitutions associated with reduced susceptibility to lenacapavir, thereby establishing a molecular baseline against which the future emergence of capsid resistance in Bulgaria can be assessed. To our knowledge, no population-based assessment of capsid variability and of substitutions associated with lenacapavir resistance has so far been reported from South-Eastern Europe. The study was therefore designed as a pre-introduction baseline assessment, analogous to the surveys of transmitted drug resistance conducted before a new antiretroviral class enters clinical use: because lenacapavir is not yet in use in Bulgaria, these data provide the reference point against which any future emergence of capsid resistance can be interpreted.

## 2. Materials and Methods

### 2.1. Study Design and Specimen Preparation

Between 2023 and 2025, a total of 842 individuals were newly diagnosed with HIV-1 infection in Bulgaria. Plasma samples from lenacapavir (LEN)-naïve individuals were collected and processed at the National Reference Confirmatory Laboratory of HIV (NRCL of HIV), National Center of Infectious and Parasitic Diseases (NCIPD), Sofia, Bulgaria. Samples were considered for whole-genome sequencing when a plasma aliquot was available and the plasma HIV-1 RNA level met the requirement of the whole-genome genotyping assay described in Section 2.2, for which the manufacturer specifies a minimum of 25,000 copies/mL; samples with lower viral loads were also processed in some instances, but amplification succeeded less consistently. This procedure yielded HIV-1 gag sequences for 115 individuals, and following quality control, 103 sequences were retained for subsequent subtyping, phylogenetic, drug-resistance, and statistical analyses, corresponding to 12.2% of all HIV-1 infections newly diagnosed in Bulgaria during the study period.

At the time of HIV-1 diagnosis, demographic and epidemiological data, including the probable route of transmission, probable country of infection, and the presence of a concurrent sexually transmitted infection (STI) other than HIV, were collected using a standardized self-administered questionnaire in accordance with applicable national regulations. To ensure confidentiality, demographic and clinical data were anonymized using unique digital codes in accordance with the applicable ethical and data-protection requirements of the NCIPD and the Republic of Bulgaria.

Blood samples were processed at the NRCL of HIV by centrifugation, after which plasma aliquots were stored at −80 °C until further analysis, as previously described [7].

### 2.2. Sequence Analysis and Data Set

Viral RNA was extracted from plasma samples using the QIAamp Viral RNA Mini Kit (QIAGEN, Hilden, Germany) according to the instructions provided by the manufacturer. The HIV-1 gag gene was amplified using the DeepChek Whole Genome HIV-1 Genotyping Assay v1 (Advanced Biological Laboratories (ABL), Luxembourg) [31]. The present study focused exclusively on the analysis of the HIV-1 gag gene; therefore, only samples with high-quality gag sequences passing quality control were included. Sequencing libraries were prepared using the DeepChek NGS Library Preparation v1 + UDI Adapters kit (ABL, Luxembourg), followed by purification with DeepChek NGS Clean-up Beads v2 (ABL, Luxembourg) [31]. Libraries were sequenced on an Illumina MiSeq System (Illumina, San Diego, CA, USA) using the MiSeq Reagent Kit v2 (2 × 250 bp) and sequencing by synthesis (SBS) chemistry. The DeepChek^®^ workflow generates three overlapping amplicons covering the HIV-1 gag region, including the regions encoding the matrix (p17), capsid (p24), and nucleocapsid (p7) proteins. The gag region was therefore sequenced as part of a whole-genome workflow already established in the laboratory; consensus sequences of comparable quality can also be obtained by population sequencing. Raw FASTQ reads were processed and assembled using Geneious Prime^®^ v2022.1.1 (Biomatters Ltd., Auckland, New Zealand). Sequence reads were mapped against the HIV-1 HXB2 reference genome (GenBank accession no. K03455.1) [32]. A minimum read-depth threshold of 1000× was applied for consensus sequence generation. Minority variants reaching a frequency of ≥20% at a given position were reported as mixtures using the corresponding IUPAC ambiguity code. This threshold approximates the detection limit of population sequencing, on which the current clinical interpretation of genotypic resistance is based; the clinical significance of capsid variants present below this level has not been established. The overlapping amplicons were subsequently aligned and assembled into a continuous gag consensus sequence. Nucleotide positions with insufficient sequencing coverage or ambiguous base calls were represented as N and were retained as such for subsequent molecular analyses.

Sequence quality control (QC) was subsequently conducted using the specialized web-based tools from the Los Alamos National Laboratory (LANL) HIV Sequence Database (https://www.hiv.lanl.gov/ (accessed on 20 September 2026)). Potential sequence anomalies, including frame-shift mutations and premature stop codons, as well as overall sequence quality and alignment integrity, were assessed using the LANL Sequence Quality Control (QC) tool. In addition, APOBEC3G/F-mediated G-to-A hypermutation was assessed using the HYPERMUT 2.0 tool [33]. To ensure the quality and reliability of the data for downstream phylogenetic and evolutionary analyses, sequences identified as hypermutated by HYPERMUT 2.0 or exhibiting a non-ACGT nucleotide proportion ≥ 3.5% were excluded from further analysis.

HIV-1 subtype and recombinant-form assignments were assessed using the web-based tools COMET v2.4, REGA v3.0, jpHMM, and RIP [34,35,36,37]. For phylogenetic analysis, HIV-1 subtype reference sequences were retrieved from the 2023 HIV-1 subtype reference alignment dataset available through the Los Alamos National Laboratory (LANL) HIV Sequence Database (https://www.hiv.lanl.gov/ (accessed on 20 September 2026)). To minimize the potential overrepresentation of individual lineages and to maintain a balanced and interpretable phylogenetic tree, one representative sequence was selected from the reference dataset for each subtype, sub-subtype, and circulating recombinant form (CRF), resulting in a total of 162 reference sequences. The LANL reference alignment is curated so that each entry is representative of its lineage, and this approach ensured that all currently recognized subtypes, sub-subtypes and CRFs were included without any single lineage dominating the alignment. The reference set comprised 17 sequences representing the non-recombinant subtypes and sub-subtypes A1–A4, A6–A8, B, C, D, F1, F2, G, H, J, K, and L, together with 145 sequences representing CRFs. Multiple sequence alignment of the LANL reference sequences and the Bulgarian sequences was performed using MAFFT version 7 and subsequently inspected and manually curated in AliView version 1.17.1 [38,39]. The final alignment comprised 265 sequences, including 103 Bulgarian HIV-1 gag sequences and 162 reference sequences.

Phylogenetic relationships were inferred using the maximum-likelihood (ML) method implemented in the stand-alone software IQ-TREE v3.1.2 (https://iqtree.github.io/ (accessed on 20 September 2026)) [40,41]. Prior to tree reconstruction, the optimal nucleotide substitution model was automatically selected using ModelFinder integrated within IQ-TREE, applying the Bayesian information criterion (BIC) [42]. Branch support was statistically assessed using the ultrafast bootstrap approximation (UFBoot2) with 1000 replicates [43].

Capsid inhibitor resistance-associated mutations (CAI RAMs) were identified using the HIVdb Program Mutation Analysis Tool, version 10.2, available through the Stanford University HIV Drug Resistance Database, with drug-resistance interpretation performed using the Sierra algorithm (https://hivdb.stanford.edu/hivdb/by-patterns/ (accessed on 20 September 2026))).

### 2.3. Statistical Analysis

Categorical variables were summarized as counts and percentages, with 95% confidence intervals (CI) calculated using the Wilson score method. HIV-1 gag lineages were classified as subtype B or non-B and compared across demographic and epidemiological strata using Fisher’s exact test for 2 × 2 tables or a Monte Carlo exact chi-square test with 10,000 permutations for larger tables, because expected cell frequencies were frequently below five. Binary logistic regression was used to evaluate independent associations of sex, probable country of infection, and concurrent sexually transmitted infection with infection by a non-B lineage. All tests were two-sided, with statistical significance defined as *p* < 0.05. No adjustment for multiple comparisons was applied; therefore, association analyses were considered exploratory. Statistical analyses were performed in Python v3.11 [44] using the SciPy v1.17 [45] and statsmodels v0.14 [46] libraries. Claude (Anthropic, San Francisco, CA, USA) was used to assist with statistical code development. All analyses and reported results were reviewed and verified by the authors against the primary dataset.

## 3. Results

### 3.1. Demographic and Epidemiological Characteristics of the Study Population

Of the 115 plasma samples available for HIV-1 gag sequencing, 12 (10.4%) were excluded during quality control, resulting in a final dataset of 103 sequences from individuals diagnosed between 2023 and 2025. Men predominated markedly, accounting for 91 cases (88.3%; 95% CI 80.7–93.2), as compared with 12 women (11.7%). The most frequently reported probable route of transmission was heterosexual contact (HET; 55/103, 53.4%), followed by sex between men (MSM; 36/103, 35.0%), injecting drug use (PWID; 9/103, 8.7%), and combined MSM and injecting drug use (3/103, 2.9%) (Table 1).

Probable infection within Bulgaria was reported for 80 individuals (77.7%), whereas 23 (22.3%; 95% CI, 15.4–31.3) reported probable exposure abroad. Reported countries of probable exposure were predominantly in Europe, including the United Kingdom, Germany, Italy, Spain, Austria, Greece, France, and the Czech Republic; individual cases reported exposure in Belarus, Türkiye, the United Arab Emirates, Canada, and the United States of America. One participant of foreign origin (Russian Federation) was reported to have acquired the infection in Bulgaria. Based on the predefined study classification, 24 infections (23.3%) were classified as imported. A concurrent coinfection with other sexual transmitted infections, including hepatitis B virus or hepatitis C virus, was reported in 31 individuals (30.1%) (Table 1).

According to permanent address at diagnosis, participants represented 21 of the 28 administrative regions of Bulgaria. The largest proportion resided in Sofia (43/103, 41.7%), followed by Plovdiv (11/103, 10.7%), Haskovo (8/103, 7.8%), Vratsa and Stara Zagora (6/103 each, 5.8%), and Burgas (4/103, 3.9%). Each of the remaining regions contributed three or fewer sequences (Table 2).

### 3.2. Distribution of HIV-1 Gag Lineages

Subtyping of the HIV-1 *gag* region using COMET v2.4, REGA v3.0, jpHMM and RIP, together with the phylogenetic analysis, identified nine distinct HIV-1 lineages. Subtype B was the most prevalent single lineage, accounting for 48 of the 103 sequences (46.6%; 95% CI 37.3–56.2). Non-B lineages collectively accounted for the majority of the study population, with 55 sequences (53.4%; 95% CI, 43.8–62.7). Among the assigned non-B lineages, F1 was the most frequent (14/103, 13.6%), followed by CRF01_AE (9/103, 8.7%), A1 and CRF02_AG (6/103 each, 5.8%), A6 (4/103, 3.9%), C (3/103, 2.9%), and CRF14_BG (2/103, 1.9%). Eleven sequences (10.7%) could not be assigned to a pure subtype or to an established circulating recombinant form with sufficient support by any of the subtyping tools and were therefore reported as unassigned (Table 3).

### 3.3. Phylogenetic Analysis

The final alignment comprised 265 sequences and 1503 nucleotide positions, including 470 constant, 864 parsimony-informative, and 169 singleton sites. The mean proportion of gaps and ambiguous characters was 2.0%, and none of the sequences failed the composition chi-square test, indicating no evidence of compositional bias. ModelFinder selected GTR + F + R7 as the best-fitting nucleotide substitution model according to the Bayesian information criterion.

The tree topology was broadly concordant with the lineage assignments obtained using the automated subtyping tools, with sequences assigned to the same lineage generally showing consistent phylogenetic placement (Figure 1). For several lineages, however, the closest reference sequence was a circulating recombinant form containing a gag fragment derived from the corresponding parental subtype. This was observed for F1 and A6 sequences, which grouped closest to BF1 and A6B recombinant references, respectively, and for subtype C sequences, which grouped closest to BC recombinant references. Nine of the 11 sequences classified as unassigned grouped in the vicinity of BF1 recombinant references, suggesting that their gag regions derive from recombinants with subtype B and F1 parental fragments rather than reflecting inadequate sequence quality.

Two closely related pairs were nevertheless apparent. The two CRF14_BG sequences, obtained from two PWID resident in the same region, formed a pair supported by an ultrafast bootstrap value of 100 and separated by a patristic distance of 0.004 substitutions per site.

The two sequences carrying the accessory capsid substitution T107S (PZ795398 and PZ795432), both assigned to CRF01_AE and obtained from men residing in the Sofia region who reported heterosexual transmission, formed a distinct pair nested within the CRF01_AE clade, supported by an ultrafast bootstrap value of 100 and separated by a patristic distance of 0.005 substitutions per site (Figure 1). The sequence carrying the T107T/S mixture was assigned to F1 and was phylogenetically unrelated to this pair.

### 3.4. Lineage Distribution by Sex, Origin of Infection and Probable Route of Transmission

Subtype B was the most frequent lineage in both men and women, accounting for 41 of 91 infections in men (45.1%) and 7 of 12 infections in women (58.3%). Sub-subtype A6 was observed in 2 of 12 women (16.7%) and 2 of 91 men (2.2%), whereas A1, C, CRF14_BG, and all unassigned sequences were observed exclusively among men (Table 3).

Among the 79 Bulgarian nationals with probable infection acquired in Bulgaria, subtype B accounted for 33 cases (41.8%), followed by F1 (12; 15.2%) and unassigned sequences (10; 12.7%). Among the 23 Bulgarian nationals with probable infection acquired abroad, subtype B was predominant (14; 60.9%), followed by CRF01_AE (4; 17.4%). The single participant of foreign origin diagnosed in Bulgaria was infected with subtype B.

The distribution of HIV-1 lineages varied across the reported transmission groups (Figure 2). Among individuals reporting heterosexual transmission, subtype B accounted for 23 of 55 cases (41.8%), followed by F1 (10; 18.2%), CRF01_AE (6; 10.9%), and unassigned sequences (6; 10.9%). Among MSM, subtype B was predominant (20 of 36; 55.6%), followed by unassigned sequences (5; 13.9%) and A1 and F1 (4 each; 11.1%). Among PWID, CRF01_AE was the most frequent lineage (3 of 9; 33.3%), followed by CRF02_AG and CRF14_BG (2 each; 22.2%), whereas subtype B was identified in 2 cases (22.2%). All three individuals reporting both MSM and injecting drug use were infected with subtype B. Both CRF14_BG sequences identified in the study occurred among PWID, whereas F1, CRF02_AG, and unassigned sequences were observed in both the heterosexual and MSM groups (Figure 2).

### 3.5. Factors Associated with Infection with a Non-B Lineage

The overall distribution of the gag lineages differed significantly according to the probable route of transmission (*p* = 0.007, Monte Carlo exact chi-square test), reflecting the concentration of CRF01_AE and CRF14_BG among PWID and the predominance of subtype B among MSM. When the outcome was collapsed into subtype B versus non-B lineages, the association with the route of transmission was of borderline statistical significance (*p* = 0.053) (Table 1). The difference between the two tests reflects the loss of information when nine lineages are collapsed into two categories: lineages with opposite distributions across transmission groups are combined, and the lineage-specific signal is thereby attenuated.

No statistically significant differences in the proportion of non-B lineages were observed according to sex (50 of 91 men, 54.9%, versus 5 of 12 women, 41.7%; *p* = 0.540), probable country of infection (46 of 80 infections acquired in Bulgaria, 57.5%, versus 9 of 23 acquired abroad, 39.1%; *p* = 0.156), imported as opposed to locally acquired infection (*p* = 0.102), the presence of a concurrent sexually transmitted infection (*p* = 0.833) or the region of residence at diagnosis (Sofia versus all other regions; *p* = 0.548) (Table 1).

Adjustment for sex, probable country of infection and the presence of a concurrent sexually transmitted infection in a binary logistic regression model did not alter these findings, and none of the variables examined was independently associated with infection by a non-B lineage. Infection probably acquired abroad was associated with a lower, although statistically non-significant, likelihood of carrying a non-B lineage (adjusted OR 0.46; 95% CI 0.18–1.21; *p* = 0.117). These findings indicate that, within the present study population, the circulation of non-B lineages was not confined to any particular demographic or exposure group.

### 3.6. Capsid Mutations and Predicted Susceptibility to Lenacapavir

All 103 gag sequences spanned the capsid-coding region and were screened for substitutions at the amino acid positions associated with reduced susceptibility to lenacapavir (L56, M66, Q67, K70, N74, A105 and T107) using the HIVdb Program: Mutations Analysis Tool version 10.2 (https://hivdb.stanford.edu/, accessed on 4 August 2026) of the Stanford University HIV Drug Resistance Database [50]. None of the major lenacapavir resistance-associated mutations, namely L56I, M66I, Q67H, K70N/H/R, N74D, A105E and T107N [27], were identified in any of the sequences analyzed (0 of 103; 95% CI 0.0–3.6).

Substitutions at position 107 were the only changes detected at a lenacapavir resistance-associated position. The accessory mutation T107S was present in two individuals (1.9%; 95% CI 0.5–6.8), and a T107T/S mixture was observed in one further individual (1.0%; 95% CI 0.2–5.3), corresponding to a combined prevalence of 2.9% (3 of 103; 95% CI 1.0–8.2). T107S is classified in the Stanford database as a polymorphic accessory mutation that has been reported in combination with other lenacapavir-associated mutations and whose effect on susceptibility has not been established. It is therefore to be distinguished from the non-polymorphic T107N, which has been selected by lenacapavir both in vitro and in vivo, confers an approximately fourfold reduction in susceptibility when present alone, and in combination with Q67H confers a reduction of approximately 60-fold [20,26,50]. Since none of the three sequences carried Q67H or any other substitution contributing to the lenacapavir penalty score, no resistance score was assigned by the HIVdb algorithm, and all 103 viruses were genotypically predicted to remain susceptible to lenacapavir; phenotypic susceptibility testing was not performed.

All three individuals carrying a substitution at position 107 were men who reported heterosexual transmission and resided in the Sofia region. None of the three reported probable exposure outside Bulgaria. The two individuals carrying T107S were infected with CRF01_AE, representing 2 of the 9 CRF01_AE infections identified in the study population (22.2%), and both had a hepatitis C virus coinfection. In the maximum likelihood phylogeny, these two sequences formed a distinct pair supported by an ultrafast bootstrap value of 100 (Figure 1), consistent with close genetic relatedness and a possible shared transmission network or common source of infection. The individual carrying the T107T/S mixture was infected with sub-subtype F1 and was phylogenetically unrelated to this pair.

## 4. Discussion

The absence of major lenacapavir resistance-associated mutations in the present cohort is consistent with the very low prevalence of such mutations reported in capsid-inhibitor-naive populations worldwide. In one of the largest analyses published to date, comprising 21,012 group M capsid sequences from capsid-inhibitor-naive individuals, none of the previously reported lenacapavir resistance mutations reached a prevalence of 0.1%. Positions 56 and 107 were the only resistance-associated positions at which variants occurred in the absence of selective drug pressure: T107S was observed in 1.0% of the sequences and T107A and T107V in 0.3% each, with no evident association with any particular subtype, whereas the five remaining resistance positions were completely conserved among circulating isolates [27]. The substitutions identified in the present study therefore fall within the narrow range of capsid variability that is known to occur naturally. A subsequent analysis of 21,646 capsid sequences retrieved from the Los Alamos database detected lenacapavir resistance mutations in only 0.26% of sequences, Q67H being the most frequent and occurring more often in CRF01_AE [51]. A more recent global analysis of 52,927 sequences from 85 countries estimated the baseline prevalence of lenacapavir resistance-associated mutations at 0.12% [52]. Comparable figures have been reported from geographically and genetically diverse settings. A prevalence of 0.14% was reported among 2031 drug-naive individuals across multiple clades [28], although the unusual variants reported at resistance positions in that study could not be reproduced in a subsequent database search [27]. In Uganda, no major mutations were detected, while minor mutations were present in 1.6% of 546 lenacapavir-naive adults infected predominantly with subtypes A1 and D; T107A was the most frequent [29]. In Taiwan, 4.5% of antiretroviral-naive individuals had mutations, again predominantly accessory or polymorphic changes, with position 107 being the most frequently affected [30]. The combined prevalence of substitutions at position 107 observed here (2.9%) falls within this range, and the fact that all of them were polymorphic accessory changes rather than the non-polymorphic T107N supports the interpretation that no genotypically detectable primary lenacapavir resistance was present in the population studied. European population-based data nevertheless remain sparse. To date, the only published study specifically investigating natural LEN resistance in a European cohort of LEN-naive individuals is the Italian study by Lazzari et al., which examined nine primary infections by ultra-deep sequencing of near-full-length HIV-1 genomes and detected no capsid substitutions associated with LEN resistance [53]. To our knowledge, the present analysis, based on 103 consecutively diagnosed individuals, therefore provides the first population-based estimate of capsid variability from South-Eastern Europe. No comparable population-based capsid survey has been published from the neighbouring Balkan countries to date. Both sequences carrying T107S belonged to CRF01_AE and formed a strongly supported phylogenetic pair. Because substitutions at position 107 have not shown a consistent association with any particular subtype [27], their co-occurrence within a closely related pair is unlikely to be explained solely by lineage-specific variation. The two observations may therefore represent a shared transmission network or common source of infection rather than independent occurrences of T107S. All three individuals with a substitution at position 107 reported probable acquisition of infection within Bulgaria, where lenacapavir has been used neither for treatment nor for prevention, which argues against prior drug exposure. However, the gag-based phylogeny cannot establish direct transmission or the direction of transmission between the individuals. Comparable observations remain rare: in an analysis of 21,646 capsid sequences, only a single transmission couple carrying a lenacapavir resistance-associated substitution, Q67H, was identified, a finding attributed to the substantial fitness cost of such substitutions [51]. Because T107S is polymorphic and its phenotypic effect on lenacapavir susceptibility has not been established, its occurrence in closely related viruses should not be interpreted as transmitted lenacapavir resistance; rather, it indicates that variation at this position can persist in circulating HIV-1 lineages without evidence of phenotypic resistance.

This observation is particularly relevant because non-B lineages accounted for the majority (53.4%) of the sequences analyzed. Experimental data indicate that most naturally occurring polymorphisms within the lenacapavir binding site do not meaningfully alter susceptibility to the drug [54], and lenacapavir retains picomolar potency against clinical isolates representing subtypes A, A1, AE, AG, B, BF, C, D, G, and H [21]. Together with the present findings, these data provide no evidence that the genetic diversity of the HIV-1 epidemic in Bulgaria is likely to compromise the activity of lenacapavir.

These results have direct implications for the potential introduction and future surveillance of lenacapavir in Bulgaria. The drug is approved for heavily treatment-experienced individuals with multidrug-resistant HIV-1, among whom emergent capsid substitutions, predominantly M66I, were detected in 8 of 72 participants in the CAPELLA trial [22,55], and has subsequently been approved for pre-exposure prophylaxis following the demonstration of near-complete protection in the PURPOSE 1 and PURPOSE 2 trials [23,24]. Because the capsid-coding region is not included in routine genotypic resistance testing, baseline information on capsid variability remains limited [27]. The present study therefore provides a molecular baseline for the Bulgarian epidemic that can support future surveillance of capsid variability and the emergence of lenacapavir-associated resistance. Incorporating capsid surveillance into the existing national drug-resistance monitoring program would require extending sequencing beyond pol, which the whole-genome workflow used here already permits, together with a common interpretation algorithm for capsid substitutions [56]. Near-full-length genome sequencing would additionally resolve the recombinant lineages that gag-based analysis leaves unassigned, and targeted in vitro phenotypic testing would clarify whether polymorphic substitutions such as T107S have any measurable effect on lenacapavir susceptibility.

A further observation concerns the 11 sequences that could not be assigned to a defined lineage, representing 10.7% of the study population. In the phylogenetic analysis, nine of these sequences grouped in the vicinity of BF1 recombinant references. This grouping should be interpreted with caution, as assignment based on gag alone cannot establish a recombinant structure and proximity to a BF1 reference indicates only that the gag fragment derives from a subtype B or sub-subtype F1 parental segment; it nevertheless suggests that these sequences represent BF1-like recombinant lineages rather than inadequate sequence quality. This finding has two implications. First, it is consistent with the substantial contribution of both subtype B and sub-subtype F1 to the Bulgarian epidemic and suggests that recombinant forms involving these lineages may be circulating in the population, as reported in other European settings where they co-circulate [57,58]. Such forms need not be imported: near-full-length analysis of BF recombinants from Italy, where subtype B predominates and F1 is the most frequent pure non-B subtype, supported local recombination between co-circulating B and F1 viruses [59], and unique recombinant forms have previously been documented in Bulgaria [5]. Second, it qualifies the distinction between subtype B and non-B sequences used in the statistical analysis. Sequences with a subtype B component elsewhere in the genome may be classified as non-B based on the gag region alone. This makes the non-B category genetically heterogeneous and may lead to an underestimation of the contribution of subtype B. Because none of the 11 unassigned sequences carried a substitution at a lenacapavir resistance-associated position, this classification does not affect the principal resistance-related finding. Nevertheless, it should be considered when comparing the subtype B and non-B proportions reported here with estimates based on other genomic regions or near-full-length genomes.

The lineages that dominate the present dataset are also those that have shaped the epidemics of the neighboring countries, which places the Bulgarian findings in a wider regional context. Sub-subtype F1, the most frequent assignable non-B lineage in this study, is the predominant lineage in Romania, where it has persisted since the pediatric nosocomial epidemic of the late 1980s and where it subsequently drove the outbreak among PWID in Bucharest from 2011 onwards [12,14]. The prominence of F1 and of BF1-like recombinants among the Bulgarian sequences is therefore consistent with the sustained regional circulation of this lineage. A comparable parallel applies to CRF14_BG, which in the present study was detected exclusively among PWID. This recombinant form emerged among PWID in Spain and Portugal, was one of the two lineages responsible for the Bucharest outbreak, and formed the largest local transmission network of the Athens outbreak, accounting for approximately half of the PWID infections sequenced during that period [14,15]. Although based on only two sequences, the exclusive association of CRF14_BG with injecting drug use in Bulgaria mirrors this pattern and suggests that the lineage continues to circulate within injecting networks across south-eastern Europe. These observations argue for coordinated molecular surveillance across the region, particularly as capsid inhibitors are introduced into national treatment and prevention programs.

Several limitations should be considered when interpreting these findings. Resistance-associated substitutions were assessed from consensus sequences generated at a minimum read depth of 1000× and a variant-calling threshold of 20%; therefore, minority variants present below this threshold may not have been detected, and the reported prevalence estimates apply to variants detectable under the sequencing and analysis criteria used in this study. With 103 sequences and no major resistance-associated mutation detected, the study excludes a prevalence above 3.6%, but had an approximately 10% probability of detecting a mutation occurring at the prevalence of about 0.1% reported globally; it is therefore not powered to detect very rare variants. Selection for sequencing was determined by specimen availability, plasma HIV-1 RNA level and the success of whole-genome amplification rather than by random sampling, so individuals with a low plasma viral load at diagnosis are somewhat under-represented; a formal comparison with the demographic profile of all newly diagnosed cases was outside the scope of this study. Naturally occurring capsid polymorphisms are not known to be associated with plasma viral load, and the selection procedure is therefore unlikely to have biased the resistance estimates. Epidemiological data, including the probable route and country of infection, were self-reported and may be subject to reporting and social desirability bias, and the study population was predominantly male (88.3%), so the lineage distribution reported here may not be fully generalizable to women. In addition, all individuals were lenacapavir-naïve, so the substitutions detected represent naturally occurring variation rather than drug-selected variants, and the phenotypic effect of T107S on lenacapavir susceptibility has not been experimentally established. Finally, the phylogenetic analysis was restricted to the gag region and to a reference set containing a single representative per lineage, which limits the resolution with which recombinant lineages can be distinguished from the parental subtypes contributing the gag fragment and precludes formal transmission-cluster inference; the closely related pairs described here should therefore be interpreted as evidence of genetic relatedness rather than proof of direct transmission.

## 5. Conclusions

The present study provides the first baseline assessment of HIV-1 gag genetic diversity and naturally occurring lenacapavir resistance-associated substitutions among lenacapavir-naive individuals diagnosed with HIV-1 infection in Bulgaria. The circulating virus population was genetically diverse, with non-B lineages accounting for the majority of sequences and substantial representation of sub-subtype F1 and several circulating recombinant forms. Phylogenetic analysis confirmed the lineage assignments and revealed that a substantial proportion of the unassigned sequences group with BF1 recombinant references.

No major lenacapavir resistance-associated substitutions were detected among the 103 LEN-naive viruses. T107S or T107T/S was observed in three sequences, but none contained additional substitutions contributing to a lenacapavir resistance score, and all viruses were therefore genotypically predicted to remain susceptible to lenacapavir. The identification of a strongly supported CRF01_AE pair carrying T107S indicates that this accessory capsid polymorphism can be present in closely related viruses circulating within the same transmission network, without evidence of phenotypic resistance.

Overall, these findings indicate a low baseline risk of clinically relevant lenacapavir resistance in the Bulgarian HIV-1 population and provide a reference framework for molecular surveillance following the introduction and broader use of lenacapavir. Continued surveillance of capsid variability, particularly in genetically diverse and recombinant HIV-1 lineages, will be important for detecting the emergence and transmission of resistance-associated variants over time.

## Figures and Tables

**Figure 1:**
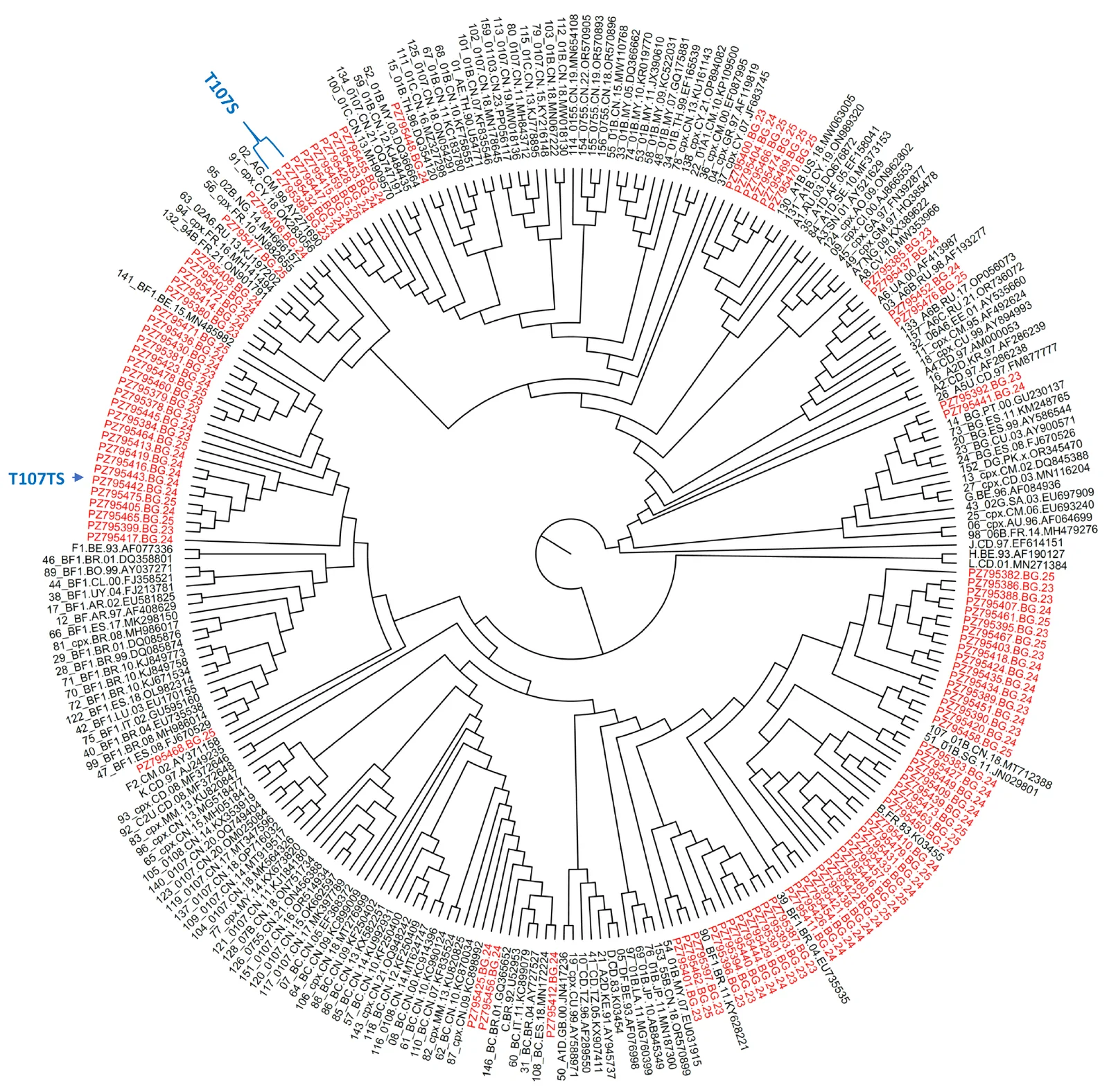
Maximum likelihood phylogenetic tree of the HIV-1 gag sequences. The tree was inferred using IQ-TREE v3.1.2 under the GTR + F + R7 substitution model selected by ModelFinder according to the Bayesian information criterion, based on an alignment of 265 sequences comprising 103 Bulgarian sequences and 162 HIV-1 reference sequences retrieved from the Los Alamos HIV Sequence Database. The tree is displayed as a circular cladogram with equalized branch lengths for visualization; therefore, branch lengths are not proportional to genetic distance. Bulgarian sequences are shown in red and labelled with the GenBank accession number followed by the two-digit year of diagnosis; reference sequences are shown in black. Blue annotations indicate the two Bulgarian sequences carrying the accessory capsid substitution T107S and the sequence carrying the T107T/S mixture. Branch support was assessed by the ultrafast bootstrap approximation (UFBoot2) with 1000 replicates; support values are not displayed on the tree and are reported in the text for the nodes discussed.

**Figure 2:**
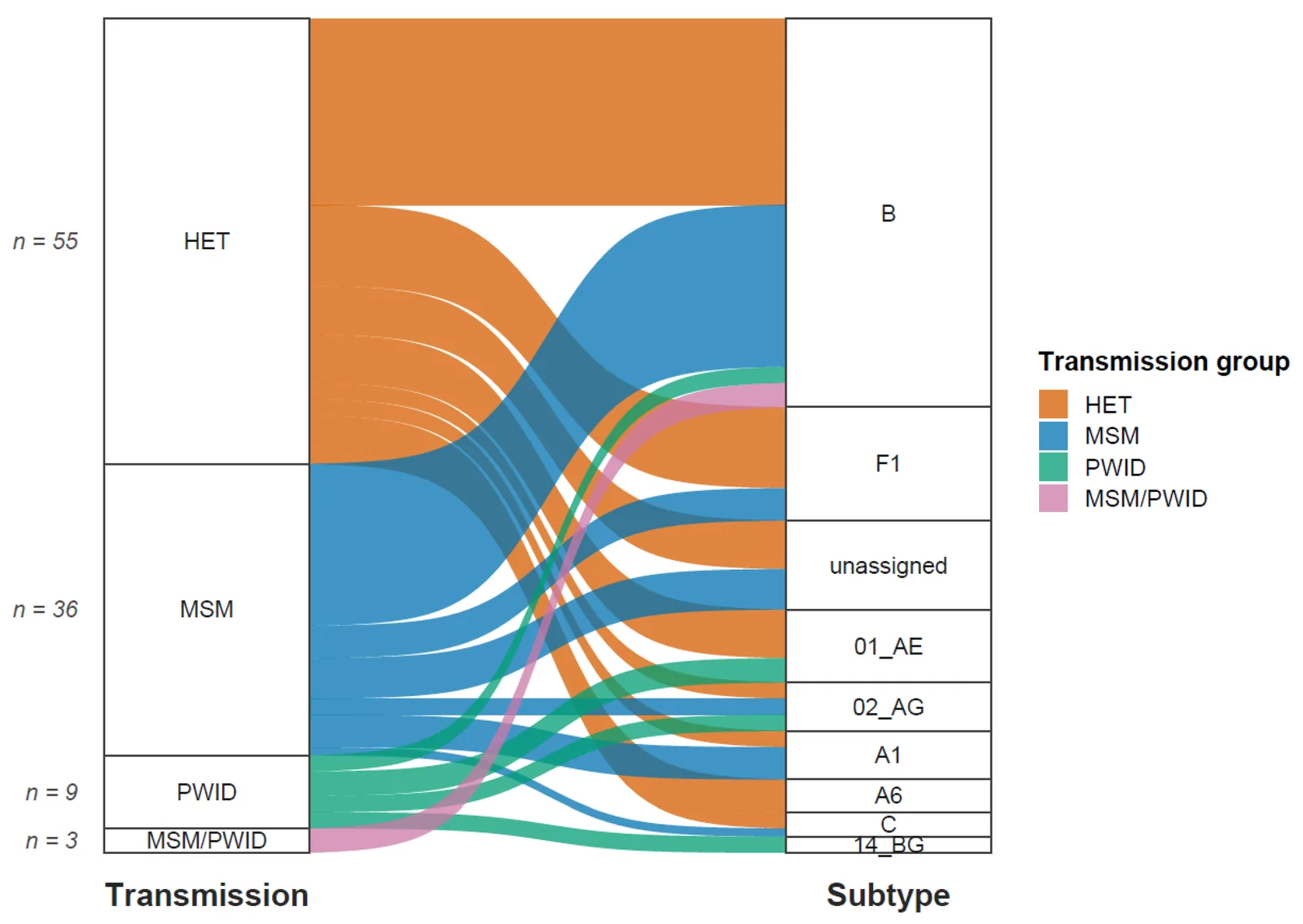
Alluvial diagram showing the distribution of HIV-1 gag lineages according to the probable route of transmission in the study population. The width of each flow is proportional to the number of sequences in each transmission group and HIV-1 gag lineage category. Unassigned sequences are shown as a separate category. The plot was generated using R v4.6.1 with the ggalluvial package v0.12.6 and ggplot2 v4.0.3 [47,48,49]. Abbreviations: MSM, men who have sex with men; HET, heterosexual transmission; PWID, people who inject drugs.

**Table 1:** Demographic and epidemiological characteristics of the study population, overall and stratified by HIV-1 gag subtype B versus non-B lineages.

Characteristic	All Sequenced Cases (*n* = 103)	Subtype B (*n* = 48)	Non-B Lineages (*n* = 55)	*p* ^1^
**Sex**				**0.540**
Men	91 (88.3)	41 (85.4)	50 (90.9)	
Women	12 (11.7)	7 (14.6)	5 (9.1)	
Probable route of transmission ^2^				**0.053**
HET	55 (53.4)	23 (47.9)	32 (58.2)	
MSM	36 (35.0)	20 (41.7)	16 (29.1)	
PWID	9 (8.7)	2 (4.2)	7 (12.7)	
MSM + PWID	3 (2.9)	3 (6.3)	0 (0.0)	
Probable country of infection				**0.156**
Bulgaria	80 (77.7)	34 (70.8)	46 (83.6)	
Abroad	23 (22.3)	14 (29.2)	9 (16.4)	
Origin of infection				**0.152**
Bulgarians infected in Bulgaria	79 (76.7)	33 (68.8)	46 (83.6)	
Bulgarians infected abroad	23 (22.3)	14 (29.2)	9 (16.4)	
Foreigners diagnosed in Bulgaria	1 (1.0)	1 (2.1)	0 (0.0)	
Imported infection ^3^				**0.102**
Yes	24 (23.3)	15 (31.3)	9 (16.4)	
No	79 (76.7)	33 (68.8)	46 (83.6)	
Concurrent STI ^4^				**0.833**
Yes	31 (30.1)	15 (31.3)	16 (29.1)	
No	72 (69.9)	33 (68.8)	39 (70.9)	
Region of residence at diagnosis				**0.548**
Sofia	43 (41.7)	22 (45.8)	21 (38.2)	
Other regions	60 (58.3)	26 (54.2)	34 (61.8)	

Values are given as *n* (%). All percentages are column percentages: those in the second column refer to the entire study population, and those in the subtype B and non-B columns refer to the 48 and 55 sequences in the respective groups, so that within each characteristic the percentages in a column sum to 100. ^1^ *p* values were obtained by Fisher’s exact test (2 × 2 tables) or by a Monte Carlo exact chi-square test with 10,000 permutations (larger tables), comparing subtype B with non-B lineages. ^2^ HET, heterosexual transmissions; MSM, men who have sex with men; PWID, people who inject drugs. ^3^ Imported infection denotes an infection probably acquired outside Bulgaria or diagnosed in a person of foreign origin. ^4^ STI, sexually transmitted infection other than HIV; this category also includes hepatitis B virus (HBV) and hepatitis C virus (HCV) infections.

**Table 2:** Geographical distribution of the gag-sequenced HIV-1 cases by region of permanent residence at diagnosis.

Region	*n*	%	Subtype B *n* (%)	Non-B Lineages *n* (%)
Sofia	43	41.7	22 (45.8)	21 (38.2)
Plovdiv	11	10.7	4 (8.3)	7 (12.7)
Haskovo	8	7.8	6 (12.5)	2 (3.6)
Vratsa	6	5.8	1 (2.1)	5 (9.1)
Stara Zagora	6	5.8	2 (4.2)	4 (7.3)
Burgas	4	3.9	3 (6.3)	1 (1.8)
Sliven	3	2.9	1 (2.1)	2 (3.6)
Pazardzhik	3	2.9	1 (2.1)	2 (3.6)
Ruse	3	2.9	1 (2.1)	2 (3.6)
Blagoevgrad	2	1.9	1 (2.1)	1 (1.8)
Vidin	2	1.9	2 (4.2)	0 (0.0)
Pernik	2	1.9	1 (2.1)	1 (1.8)
Varna	2	1.9	0 (0.0)	2 (3.6)
Gabrovo	1	1.0	0 (0.0)	1 (1.8)
Veliko Tarnovo	1	1.0	0 (0.0)	1 (1.8)
Kardzhali	1	1.0	0 (0.0)	1 (1.8)
Montana	1	1.0	1 (2.1)	0 (0.0)
Targovishte	1	1.0	1 (2.1)	0 (0.0)
Smolyan	1	1.0	0 (0.0)	1 (1.8)
Shumen	1	1.0	0 (0.0)	1 (1.8)
Kyustendil	1	1.0	1 (2.1)	0 (0.0)
Total	103	100.0	48 (46.6)	55 (53.4)

Values are given as *n* (%). All percentages are column percentages: those in the third column refer to the entire study population, and those in the last two columns refer to the 48 and 55 sequences in the respective groups. The comparison of the Sofia region with all other regions with respect to subtype B versus non-B lineages was not statistically significant (*p* = 0.548, Fisher’s exact test).

**Table 3:** Distribution of HIV-1 gag lineages according to sex, origin of infection, and probable route of transmission.

HIV-1 Lineage	Total (%)	Men (%)	Women (%)	Bulgarians Infected in Bulgaria (%)	Bulgarians Infected Abroad (%)	Foreigners Diagnosed in Bulgaria (%)	HET (%) ^1^	MSM (%) ^2^	PWID (%) ^3^	MSM + PWID (%) ^4^
B	48 (46.6)	41 (45.1)	7 (58.3)	33 (41.8)	14 (60.9)	1 (100.0)	23 (41.8)	20 (55.6)	2 (22.2)	3 (100.0)
F1	14 (13.6)	13 (14.3)	1 (8.3)	12 (15.2)	2 (8.7)	—	10 (18.2)	4 (11.1)	—	—
Unassigned ^5^	11 (10.7)	11 (12.1)	—	10 (12.7)	1 (4.3)	—	6 (10.9)	5 (13.9)	—	—
CRF01_AE	9 (8.7)	8 (8.8)	1 (8.3)	5 (6.3)	4 (17.4)	—	6 (10.9)	—	3 (33.3)	—
A1	6 (5.8)	6 (6.6)	—	6 (7.6)	—	—	2 (3.6)	4 (11.1)	—	—
CRF02_AG	6 (5.8)	5 (5.5)	1 (8.3)	5 (6.3)	1 (4.3)	—	2 (3.6)	2 (5.6)	2 (22.2)	—
A6	4 (3.9)	2 (2.2)	2 (16.7)	3 (3.8)	1 (4.3)	—	4 (7.3)	—	—	—
C	3 (2.9)	3 (3.3)	—	3 (3.8)	—	—	2 (3.6)	1 (2.8)	—	—
CRF14_BG	2 (1.9)	2 (2.2)	—	2 (2.5)	—	—	—	—	2 (22.2)	—
Total	103	91 (88.3)	12 (11.7)	79 (76.7)	23 (22.3)	1 (1.0)	55 (53.4)	36 (35.0)	9 (8.7)	3 (2.9)

Values are presented as *n* (%); percentages represent the proportion of each subgroup accounted for by the respective HIV-1 gag lineage. ^1^ HET, heterosexual transmission; ^2^ MSM, men who have sex with men; ^3^ PWID, people who inject drugs; ^4^ MSM + PWID, men who have sex with men who also inject drugs; ^5^ Unassigned, sequences that could not be classified with sufficient support by any of the subtyping tools used. Because subtyping was based on the gag region alone, this category is not equivalent to a unique recombinant form. Em dashes denote the absence of observations.

## Data Availability

The 103 HIV-1 gag sequences generated in this study have been deposited in GenBank under accession numbers PZ795378–PZ795480. All other data supporting the findings of this study are contained within the article; further enquiries can be directed to the corresponding author.
